# Book Reviews

**Published:** 1813-06

**Authors:** 


### CRITICAL ANALYSIS
### *OF RECENT PUBLICATIONS*
### IN THE
### DIFFERENT BRANCHES OF PHYSIC, SURGERY, AND MEDICAL PHILOSOPHY.

*Cases of Hydrophobia; including* Dr. Schoolbred's *and* Mr. Tymon's *successful Cases; with some Observations on the Nature and Seat of the Disease. By* J. O'Donnel, M.D. 8vo. pp. 63. Callow. Lond. 1813.

TWO cases of Hydrophobia are here related by Dr. O'Donnel, as having come immediately under his own notice: and they are valuable as adding to the stock of facts arising in a morbid condition of the system with which we are but little acquainted; which is still mysterious, and we much fear yet unmanageable. They both occurred in the practice of the author, within a few months; both had a fatal termination; and with one of them was connected a circumstance unusual, if not new. But that we may not withhold from the faculty the facts here stated, or hazard a misrepresentation by an abridgment, we shall reprint the cases in the words of the author.

"Case I.—William Honey, aged 35, a bargeman, working on the Grand Junction Canal, had a puppy three months old. On Sunday, the 22d of December, 1811, whilst the barge lay at Paddington, the animal eat an ounce of tobacco, belonging to his master, which he found in the barge, and ran away, nobody knew where; but returned at night, and did not appear to be ill. On his return, his master, master, who was very fond of him, began, as it was his custom to do, to play with him; he pinched him and pulled him about, to make him sharp, as he called it; the puppy snarled and snapped a little, and one of those snaps slightly scratched the skin, about a quarter of an inch on the back of his master's right hand. On the same night he eat another paper of tobacco, about an ounce, and the following morning early he ran away again, and was not afterwards heard of. Two days before he bit Honey, he bit another bargeman in play; and the day after this he bit Honey's boy, a lad seventeen years of age, in both hands, slightly, also in play. The dog's teeth entered, and drew blood, but the parts got well immediately. The scratched part of Honey's hand had a high scab upon it, which did not fall off for about a fortnight, at which time it was quite well. The puppy being generally confined to the barge, was seldom on shore, and could not, by the statement of the barge people, have been bitten by another dog. It is reasonable, therefore, to conclude that, that state of diseased secretion, a portion of which, when introduced into the human body, frequently produces hydrophobia, originated in the dog itself. Whether eating the tobacco, could have had any thing to do with its production, I am not able to form a conjecture. No alteration in the dog appeared, except that he looked thin.

" The other bargeman had never felt any bad consequences from the bite; and the boy, Robert Rose, I have frequently seen since, and he keeps perfectly well.

" The foregoing facts I have taken from Honey and his wife; the following after his death from his wife, who, during his illness, lived on-board with him.

" Honey continued at his work, as barge-master, until Thursday, the 23d of January, thirty-three days after the bite. On this day he complained of wind and pain in his stomach, with shortness of breath, and of being much purged; complaints to which he was not accustomed. He continued on-board his barge, and had a very restless night without any sleep; during the night he complained of pain under the right ear. On Friday, the 24th, he looked very ill, and complained of soreness in his throat. The purging and shortness of breath continued. He drank tea in the evening. On the night of this day he complained of a violent, and to use the woman's words as employed by him, an uncommon pain, such as he never felt before, in his shoulder. His purging had ceased from the evening before. He passed a very restless night, and had a rigor, which he compared to similar feelings which he had been accustomed to experience whenever he caught a heavy cold. It was of short duration, and his skin, after it was over, became hot, but not in any very great degree; his breathing got worse. On Saturday morning these symptoms continued, with little perceptible alteration, except in his countenance, which looked very dejected. He heretofore shewed no dislike to drink; this evening he drank a little table beer, on which he made no observation, and went to bed, complaining much of the pains in his throat and shoulder; but of the shortness of his breath he complained more than of the pains. He passed a very bad bad night, frequently jumping out of bed, being afraid, as he expressed it, of being strangled. He said he felt a sensation in his breathing as if he had been suddenly thrown into cold water. Sunday, the 26th, in the morning, he left his barge, and came to his lodging in Uxbridge. Expressing a hope that bleeding would relieve his breath, he went to Mr. Rayner's of Uxbridge, where about fourteen ounces of blood was taken from his right arm by Mr. Rayner's assistant. He said nothing about his complaint to the gentleman who bled him. He walked home without any faintness from loss of blood, his wife accompanying him. After he got home, she discovered that he could not drink; on putting a tea spoonful of tea to his lips, he drew back his head with great horror. He made several attempts to put it in his mouth, but as often drew back his head with great precipitation and terror. He became much alarmed, and talked seriously of his affairs to his wife; but never mentioned the bite, nor appeared to suspect that it had any thing to do with his present indisposition. He went to bed, but did not sleep, nor had he slept one minute since the first symptom of attack. His pains continued in much the same state, and he complained constantly of the catching of his breath, as if plunged suddenly into cold water. Since Friday, when the diarrhœa stopped, and the pain attacked his shoulder, he had no alvine evacuation. On Monday, the 27th, he expressed a wish to have medical assistance, and Mr. Rayner was consulted, who prescribed what is called an antispasmodic mixture for him. At ten o'clock, on the same day, he wished to see me. I found him sitting up in bed, his wife and another woman holding his hands, reading prayers to him, to which he was frequently, and irregularly, exclaiming, Amen; evidently under a degree of mental distraction, accompanied with that most horrid expression of countenance peculiar to the hydrophobia. On taking hold of his hand, I found him in a clammy sweat, with a pulse so irregular that it could not be told. He said he had taken a bad cold, and had the rheumatism in his arm, shoulder, and neck, and had a very bad sore throat. I encouraged him much, by agreeing with him in his own opinion of his case; but having seen cases of hydrophobia before, I had no doubt of the true nature of his complaint. I expressed a hope that he would get better soon; this, although it suspended his terror, did not long keep off his agitation, which was rapidly advancing to a height seldom, even in such cases, observed. At this moment he seized hold of my hand in his, which he grasped with a vice-like force, and from which, with much difficulty and some assistance, I got disengaged; his eyes, at this time, darting at the moving objects about him, with a firmness, and fierceness, and quickness, beyond all language to describe. In a few minutes the paroxysm subsided. As I found he had been talking without concern of the bite of the dog, I questioned him concerning the fact. He related to me, with the greatest precision, the facts above stated; although evidently with some reluctance, accompanying his story with—" but the puppy was not mad, he only eat some tobacco."—On looking at the part bitten, there appeared a circular redness, which at times disappeared, and came again—there was no hardness to be felt, nor cicatrix to be seen—a small ash-

 colored colored speck, scarcely visible, was all the eye could discover in the absence of the redness. I desired him to drink a spoonful of the medicine he had been supplied with; the bare mention of which threw him into one of those hydrophobic sobbings, and distortions of countenance, peculiar to this disease. I continued a long time soliciting him to drink, but without being able to prevail upon him, even to make an attempt. At length, with a wet feather, I touched his lips, which threw him into the most violent agitation. Knowing that no advantage could be derived from further efforts of this sort, I discontinued all attempts and retired, advising his friends to lose no time in getting a strait jacket on him; as I supposed, from the strength of the man, and the excessive violence of the paroxysms, that mischief might arise, and without this mode of confinement, no attendants could keep him quiet. I had not left the house many minutes before a paroxysm came on, in which he laid hold on his wife, who sat on his bed with him, threatening her life, declaring they should die together; and she thinks he would have killed her, had she not been disengaged by several men who were in the room at the time. In his violent effort to accomplish this object, the vein, which had been opened on the morning of Sunday, burst out afresh, and a considerable quantity of blood flowed, before any person could be found to stop it. Mr. James, the surgeon in Uxbridge, being procured, bound up the arm, and left him apparently dying with loss of blood. On being apprised of this circumstance, I returned to his apartment, and found him recovering from the faint state to which the loss of blood had reduced him. Soon after I got to his bed-side, another paroxysm came on, in which he arose to nearly an erect posture, when he suddenly fell backwards in bed with a quick spasmodic violence, such as a person laboring under an attack of trismus might be supposed to do. His head and body were evidently pulled backwards by the rigid state, or spasmodic action, of the muscles of the neck and back. At this time he pumped up a great quantity of white foam resembling soap lather. The strait jacket, although procured, was not put on. At least a dozen men were in the room, but they were afraid to approach him. A soldier, however, who had seen the jacket used before, had courage, between the fits, to put it on. The patient sat up whilst this was doing, hurrying the person who sat behind him tying it, to make haste and let him lie down. On the soldier quitting his seat, his body returned to the bed with convulsive quickness and force, and he again brought up a quantity of foam. His abhorrence to fluids, or food of any kind, continued. The disease was too far advanced to try medicine, and as I had no faith in any remedies heretofore used, I gave it as my opinion that there was nothing to be done, but to watch the termination of the case, which I thought could not last many hours. Mr. Rayner conceiving that a dose of opium and camphire might lessen the excessive irritability, attempted to give him a bolus composed of those ingredients, which the poor fellow with eagerness opened his mouth to receive; but on the spoon getting into his mouth, he snapped his teeth upon it, and held it fast for some time, saying, when it was withdrawn, "I thought I had caught you." I saw him again in the evening, when the violence of the disease was was evidently abating, for the patient was sinking. He was now under a delirium, similar to that seen in the last stage of typhus.

" About eight o'clock at night he died, nearly five days from the commencement of his illness, and thirty-nine hours after hydrophobia appeared.

" I am indebted to Mr. Copeland, of Golden Square, who had business in the neighbourhood at that time, for the following description of the appearance of the body upon dissection.

" *Dissection.*—January 28. Tuesday evening, in the presence of Dr. O'Donnel, Mr. Rayner, Mr. James, and Mr. Bingham, surgeons, I opened the body of William Honey, who had died the preceding day of hydrophobia.

" The surface of the whole intestinal canal, but particularly of the small intestines, exhibited evident marks of inflammation. When the stomach was opened, there was the appearance of unusual vascular action; but in three or four spots, about the size of a sixpence, the internal coat of the stomach was discolored, of a brown, or approaching to black color. That I might not be deceived in this appearance, I requested the gentlemen present to observe it particularly, and it was evident that these spots had undergone a very material change of structure, that they were in a state of sphacelation. When I took out the larynx, pharynx, and part of the tongue, for more accurate examination, the epiglottis presented itself with its membrane very highly injected with red blood, as was also the membrane covering the constrictor muscles of the pharynx, and generally the whole of the larynx was very much inflamed. These parts are preserved by Mr. Rayner. The vessels of the dura mater, and pia mater, were turgid; and the lateral ventricles contained, I suppose, about four ounces of fluid; but the principal appearance of inflammation in the brain was at the base, near the crura cerebri, the tuberculum annulare, and the origin of the eighth pair of nerves; the pia mater was here considerably more vascular than in any other part, and there was also much fluid effused under it. A particular red appearance of the part, where the wound had been inflicted, was observed before death; but nothing could be detected on inspection, except that a considerable branch of the cutaneous nerve was traced to the wound; this nerve was laid bare for some distance, but no inflammation or change of appearance was manifest. T. Copeland."

" Case II.—Joseph Watson, of the parish of Jekenham, near Uxbridge, aged four years, was bit in the cheek by a bull-bitch, who had puppies a month old. The child, together with the children of Mr. Payne, a farmer, the master of the bitch, were in the constant practice of playing with her and her puppies, as Watson lived in a cottage adjoining Mr. Payne's.

" On the same day that Joseph Watson was bit, the bitch, at play with the children, rolled upon her back in a fond manner, and licked the face of one of them, apparently in her usual health and temper. The day before this she was observed to bite a cow, who had got into the stable in which her puppies were, and afterwards she bit a hog in the yard. Her master correcting her for these offences, observed that she humbled herself to him, and lay down, as dogs do when undergoing chastisement, and shewed not the least appearance of disease. She ate and drank as usual, and looked plump and well. Having bitten the child, notwithstanding her correction the day before, her master determined to destroy her, as a vicious animal, and called one of his servants to assist him in the execution of this resolution. She made no resistance, although they shook her, and scolded her much. Mr. Payne held her, his man fastened the rope, and without any attempt to bite either of them, she was destroyed.

"The child was brought to my house in a cart, on the 19th of August, 1811; he had been bitten a month before, wanting one day. The cicatrix looked red, but was perfectly healed. As there was no suspicion of the dog, no attention was paid to the wound; a common dressing was all the remedy used. On Wednesday the 14th of August, his mother said he complained of sickness of stomach, and giddiness of head; he was frequently sick, but did not vomit. On Thursday his belly was swelled, and very hard, and he was a good deal purged, which she supposed arose from his having eaten plumbs; but she does not know that he ate any. Friday his bowels continued loose, passing thin, foul matter. He was very dull, and did not like to speak or get out of bed. On Saturday his purging ceased, and he complained of sore throat. On Sunday morning his father and mother became terrified at his appearance, and observed that he could not drink his tea at breakfast. On Monday morning they brought him to me. His first appearance discovered to me that the disease under which he laboured was hydrophobia, and of which the poor woman had no suspicion. My first question produced the discovery that he had been bitten by a dog, but she said it was not mad. I offered him a little water to drink, which produced the usual symptoms of horror and suffocation. Offering him the empty cup had the same effect. It rained at the time, and I had him taken out and exposed to it, without producing any distress, save that which moving him in the air produced; for when he was kept out of motion in the rain, he was calm, and as soon as he was put in motion, he began sobbing and struggling, with that characteristic countenance which this disease only exhibits.

"I knew of no remedy, and recommended nothing but laudanum, which they could not get him to take. He died in the evening, having been ill six days from the first attack, and having had the symptoms of hydrophobia thirty-five hours.

"As this was the first case of hydrophobia which had occurred in this neighbourhood, of late years, I mentioned it to the medical gentlemen of Uxbridge, all of whom, particularly Mr. James, gave their attendance.

"A few days after the death of the boy, the rector of the parish, who was greatly interested in the fate of the child, informed me that the cow and the hog were mad. Never having seen animals in this state, I called to see them. The cow was evidently mad, and very mischievous. The hog appeared to be stupid or foolish. They were both destroyed."

A re-

A remarkable circumstance, connected with the case of Joseph Watson, was that the bitch by which he was bitten showed no marks of disease, and she was destroyed, not on suspicion of being rabid, but because she was considered as a vicious animal. It is still further remarkable that all the animals bitten by this bitch, though she herself was free from the appearance of disease, became rabid. The child, Joseph Watson, died 35 hours after hydrophobic symptoms appeared; the cow was evidently mad; and the hog appeared to be stupid or foolish. In all other instances, and where the dog has been evidently under the disease, the greater number of the bitten have escaped.

A very considerable portion of the pamphlet is employed in the detail of Dr. Schoolbred's and Mr. Tymon's cases, both of which having previously appeared in the pages of our Journal, we shall pass over, to notice some opinions of Dr. O'Donnel, on the treatment and phenomena of the disease. To the state of the stomach and bowels much importance is attached.

"The affection of the stomach in Amien's case is described by Dr. Schoolbred thus:—'Whenever he could get his hand disengaged, he immediately struck the pit of his stomach with it, pointing out that part as the seat of some indescribable uneasiness.' And again, as before stated,—'He put his hand on the region of his stomach, and said that the pain in that part was returning;' and during the second bleeding the pain in his stomach ceased. In the case of Honey, the first symptoms of the disease were wind and pain in the stomach, with diarrhœa; and it was not until constipation took place, that he had the symptoms of hydrophobia. In Joseph Watson's case, pain in the stomach, sickness, giddiness, tumid, and tense abdomen, followed by diarrhœa, continued; as was also the case with Honey, some days before hydrophobia came on; and in this case of Watson's, constipation also took place before this characteristic symptom appeared. In both cases hydrophobia did not shew itself until the sore throat or inflammation of the larynx, pharynx, &c. came on.

"The dissection of Honey's stomach shewed marks of high vascular action; and in places marked by dark spots, sphacelation. In the unsuccessful case of Mr. Tymon, the morbid appearance of the body was almost confined to the stomach; which he describes as being inflamed, and that blood was extravasated from several of its vessels. It was also loaded with bile.

"In what way the poison introduced into the human body, by the bite of a rabid animal, disturbs the functions, and produces the disease of the stomach and bowels, we have yet to learn: the fact, however, is beyond all doubt.

"That the nature of the disease is not spasmodic, is proved, as far as any thing can be proved, by experience. In no disease has there been a more free use made of opium, camphor, and all the class

 of of medicines denominated antispasmodics, than has been made in this, without the instance of a single cure having been effected by them. That it is a highly inflammatory disease, affecting the stomach in the first instance, and extending from it to the larynx, pharynx, œsophagus, &c. is, I think, probable; and, in support of this opinion, in addition to the evidence we derive from dissection, in numerous cases, the cure of Dr. Schoolbred's case was effected by the only means we know of for curing inflammatory disease, viz. by bleeding, purging, and vegetable diet."

On copious and bold bleeding at the first appearance of disease, great expectation is founded; and to this, with an unloading of the stomach and bowels by purging, we are taught to look forward with the hope of a successful treatment of a malady, which has so generally, if not always, when specific, baffled human skill. From the appearance of inflammation about the digestive organs, which dissection had so often manifested does take place, we admit the ground of this treatment to be most rational; and we still remain sceptical as to its success.

---

*The Accidents of Human Life, with Hints for their Prevention, or the Removal of their Consequences. By* NEWTON BOSWORTH. 12mo. London, 1813. pp. 209. Plates.

THE benevolent intention of this little volume would claim indulgence from the critic, if faults in it did require indulgence. As a popular directory on various occasions, and it professes to go no farther, we freely recommend it. The 2d, 3d, 4th, and 5th parts, contain the author's remarks on accidents from fire, with directions for escaping from burning houses—account of *fire-escapes*—instructions for extinguishing fires—compositions to extinguish fire—danger from burning clothes—modes of guarding against fire. The 6th, 7th, and 8th parts, treat on accidents from water. In these are given descriptions of the means of raising bodies from the water—of drags—means of recovering persons apparently drowned, or suffocated—some account of the Humane Society—dangers of the sea—shipwrecks—life-boats—cork-jackets—life-preservers—and Lieutenant Bell's and Captain Manby's methods. The 9th part comprehends those accidents which occur in dangerous sports, from falls, bites of dogs, wounds, burns, gunpowder, and fire-arms. It contains likewise a description and plate of Colonel Crichton's bed and frame for removing wounded persons. The 10th and last part is on accidents occurring in travelling—intense cold—sudden changes from cold to heat—thunder storms—

fainting—

fainting—and cautions against "indulging in extreme *sensibility.*"

The scope and object of this volume will hardly admit of any extract being made from it that will interest *our* readers. The following short statement of substances that have been dissolved in or mixed with water, with the view to increase its power of extinguishing fire, may not be without interest.

"Water and fire have long been accounted enemies; and it is in consequence of this enmity that the former is always resorted to whenever the latter is likely to do mischief—indeed it is almost the only ingredient which is thought of, in general, when we want to put out a fire. It has often been a question, however, whether some other things might not be thought of, which, by being mixed with water, or dissolved in it, would render it still more eminently useful. Several persons have turned their attention to the subject, and with some degree of success.

"Among others, Mr. William Knox, of Gottenburg, in Sweden, made some experiments with compositions for this purpose. He divides them into simple and compound solutions. Of the latter sort, which he prefers as the surest and most powerful, I shall give you a few examples.

| | | |
|---|---|---|
| 1. | Water . . . . . . . . | 75 galls. |
| | Clay . . . . . . . . | 10 qts. |
| | Vitriol . . . . . . . . | 10 do. |
| | Common Salt . . . . . . | 10 do. |
| 2. | Water . . . . . . . . | 75 galls. |
| | Strong solution of wood ashes . . . . | 18 qts. |
| | Fine clay reduced to powder . . . . | 18 do. |
| 3. | Water . . . . . . . . | 75 galls. |
| | Red ochre, or the residuum of aquafortis . | 15 qts. |
| | Common salt . . . . . . | 15 do. |
| 4. | Water . . . . . . . . | 75 galls. |
| | Strongest herring pickle . . . | 15 qts. |
| | Red ochre . . . . . . . | 15 do. |

"That these mixtures, or indeed almost any other which will render the water more dense without much decreasing its fluidity, would put out a fire more speedily than water alone, is very likely, since it is principally by covering the burning body and keeping from it the air which would feed the flame, that water itself is so useful for this purpose. There may be other qualities, however, of a chemical nature, which may render some things much more suitable to be mixed with water than others; and it is only experiment that can determine, with sufficient accuracy, which are absolutely the best. It would not be difficult to make such experiments on a small scale; and, as I really think the subject is of importance, and may prove useful, I shall be happy to assist you in the pursuit, whenever we have opportunity.

"The following is the preparation of M. Von Aken, which I give you you on his authority, as quoted in the *Pantologia,* and which appears from his account to have been eminently successful:

| | |
|---|---|
| Burnt alum . . . . . . . . . | 30 lbs. |
| Green vitriol in powder . . . . . | 40 |
| Cinabrese or red ochre powdered . . . . | 20 |
| Potter's or other clay, finely pounded and sifted | 200 |
| Water . . . . . . . . . | 630 |

With *forty measures* of this liquor, an artificial fire, which would have required the labor of *twenty men* and *fifteen hundred measures* of common water, was extinguished, under the direction of the inventor, by *three persons* only. The price of this compound solution is estimated at one halfpenny per pound.

"If such be the case, surely it would be worth while to keep in the fire-offices a quantity of the most approved ingredients, laid up in proper proportions, that on the first alarm of fire they might accompany the engines without delay, and be considered as necessary a part of the extinguishing apparatus as the engine itself.

"Other mixtures have been, at different times, proposed by various persons. Some have recommended the strewing of sand or mould upon the burning or heated parts; and when this can be done with convenience, and in sufficient quantity, it might have its use.

"Several years ago, a Dr. Godfrey, improving upon the hint of Mr. Greyl, a foreigner, tried a curious scheme for putting out fires, when they had not extended themselves beyond the room in which they began. He constructed a number of wooden vessels, which he filled with water, oil of vitriol, and sal-ammoniac: these, being thrown into rooms that were purposely set on fire, burst, after the manner of bombs, and scattering their contents by the explosion, very speedily and completely extinguished the flames. This contrivance appears better adapted to ships than houses. It does not appear to have been at all in use lately, or even to have been tried since the inventor's experiment."

---

*Tirocinium Medicum; or a Dissertation on the Duties of Youth Apprenticed to the Medical Profession. By* William Chamberlaine, Surgeon. 12mo. Lond. 1812; printed for the Author, 29, Aylesbury-street. pp. xxiv. 253.

This ingenious and lively little volume is the production of an active and intelligent practitioner, well known to the profession as the author of a useful practical work on the *Stizolobium.* After many years of experience, he has given to the public, at a low price, what has probably been dearly bought by himself. His materials are disposed in twenty-three chapters, containing advice to parents and guardians, on bringing up their children or wards to the medical profession—hints to the master—information to the candidate for apprenticeship—on apprenticing, and previous examination—

nation—abstracts of some laws of the Corporation of Apothecaries of the city of Dublin—of the prescription book, ledger, message, retail, and price books—method—primary duties—of general conduct—cleanliness—right and wrong—mistakes in making up medicines—of the manner of sending out medicines—of powders and labels—of being absent—having too many things in hand at one time—fines—of the acts of parliament imposing duties on quack medicines. An appendix, and a vocabulary.

As a specimen, we insert the first chapter, addressed "to parents and guardians;" it contains just and important observations.

" 'When you reflect on the vast importance of the science of medicine to mankind, and on the mischiefs which may ensue from the errors of an ignorant practitioner, you cannot be disposed to place your son in the profession, unless you are confident he is equal to the arduous duty. You will, I trust, not think of placing him in a situation so loaded with most serious responsibility, unless you are satisfied that his mental abilities are such as give a fair prospect of his obtaining those acquirements by which he may be enabled to practise the art of healing to the greatest extent of advantage which the state of medical and chirurgical science will admit.'—*Parkinson's Hospital Pupil*, p. 5.

"It is no uncommon thing for parents, dazzled with the sight of so many medical men riding in their carriages—or, determined (holding *trade* in *contempt*) that a son shall be brought up to a *genteel* profession, to destine one or more of their sons, at a very early period of life, to the medical profession, without taking into consideration whether the boy, when he comes to be of proper age to be an apprentice, may like the business, or whether he has talents and qualifications for it.

" 'Could Parents be made sensible of the permanent estimation of literary and professional rudiments, or Students foresee the lasting reproach of *ignorance*, the former would need no further spur to incite their vigilance, nor the latter fail to embrace the advantages offered for their improvement. The first reflection of some parents has been to select a professional master, without any examination how far their sons have been prepared for the situation of an apprentice.

" 'A parent who would wish a young man to follow the profession of a surgeon or apothecary, with credit or commendable emulation, should take a very early survey of the requisite school-learning, as well as competency of a professional preceptor; and should not fail to count the costs of subsequent studies.

" 'In the selection of a master, it is no less essential that he should be renowned for his integrity, and a strict regard for the honor of his profession.

" 'It might be very pernicious to a young man, if the interval between leaving school and commencing an apprenticeship, were of long continuance; for, at the time of adolescency, the disposition will be active, and, by the partial indulgence of parents, a propensity to idle or bad habits may be easily acquired. During such vacation, a sedulous parent may expatiate on the charges already incurred, and those to be expected in procuring a suitable education, the advantage of turning such opportunities to profit, and the unavoidable disgrace that must be the consequence of indolence or want of application. Economy in dress, or other trifles, may be urged, by pleading the use of expending the money in more lasting professional attainments.

" 'The choice of proper companions may be another fit topic at such a season; since a particular kind, once preferred, however erroneous the choice may have been, will be afterwards with difficulty changed. The nature of the profession demands a grave deportment, and the exigencies of it oblige young men to submit to greater confinement than is required in other apprenticeships.

" 'To a failure in such necessary and seasonable inquiries, may be imputed the ignorance of many adventurous practisers.

" 'When parents have placed their children under proper teachers, they are too apt to neglect exacting obedience, or assisting in the correction of bad habits, whereas they might often animate proficiency, and preclude irreparable misconduct. Parents are not always adequate judges of the literature necessary to qualify an apprentice to a medical practitioner; but masters who are, or ought to be well acquainted with the requisite rudiments, should be cautious of admitting illiterate youth, incapable of being safely trusted to compound medicines from Latin prescriptions.

" 'Unless the health as well as capacity of a boy be duly regarded, his studies may be materially retarded, yet the most unhealthy or weakest son has, sometimes, been selected for a medical apprentice; as if indisposition were no obstacle to learning, or that employment liable to harassing fatigue, to untimely calls, and contagious diseases, were suited to a distempered frame! As well might it be argued that the smell of drugs can repair a feeble constitution, or that an apprentice may be a complaining invalid instead of an active assistant: to become master of science, and bear the drudgery of business, strength of body and activity of mind are indispensable.

" 'Every youth should first be thoroughly instructed in his native tongue, by parsing, and being grounded in grammatical rules; for one who remains ignorant in these rudiments, may reasonably be expected to be no less deficient in more difficult attainments.

" 'A clear understanding of that language in which prescriptions are commonly written, must be obviously necessary for every medical student employed in compounding medicines, and executing directions, prescribed in Latin. An ability to expound abbreviated characters, and to comprehend their true meaning, cannot be dispensed with but at the risk of fatal consequences.

" 'Many technical terms in the science are derived from the Greek language: hence the comprehension of intricate and compound names is much facilitated by a knowledge of this element. A student in medicine will more easily ascertain and remember the titles of maladies, dies, and a pupil studying anatomy can scarcely forget the situation, attachment, figure, or use of a muscle, borrowing its appellation from that language; nor will the advantages which a practitioner will receive from such a qualification be inconsiderable.'—*Lucas*.

"For, whatever line of life a parent may destine his son, the writing a good hand is so *indispensably* necessary in many businesses, and so ornamental and useful in all, that parents ought to pay particular attention towards the perfection of their children in this useful attainment. In the business of an apothecary, it is not absolutely necessary that a young man should write like beautiful copper-plate writing; but still, as he will be expected to keep his employer's books, he ought to write a neat, clean, free, and *perfectly legible* hand, both for the books, and for writing labels; for, how disgraceful is it both to master and apprentice, to see labels and directions sent to patients written in so shameful, slovenly, and careless a manner, with blots and bad spelling, as to cause patients to be afraid of taking the medicine, or oblige them to send it back to have the writing explained. Besides it may so happen that he may be called upon to manage a correspondence with some of his employer's patients, or others, and in that case how necessary is it that he should be able to be perfect in grammar and orthography, as well as good writing.

" As it will be expected, as I have just now observed, that an apprentice to an apothecary shall keep the books and write out the bills, the absolute necessity of being well versed in the common rules of arithmetic must be evident. 'A good arithmetician finds the attainment of it of so much advantage in common life, and it may prove so beneficial to a professional man, that such an acquisition ought not to be overlooked or disregarded. It is the rudiment of mathematics; and although few medical pupils have leisure for making any considerable advances, yet there is no doubt of the benefits capable of being derived from this adscititious accomplishment.'—*Lucas*, sec. 9. p. 7.

"'To sum up all, it is a debt due, not only to your son, but to the circle in which he may move, that his capacity, his education, and his disposition, be fully inquired into, and approved, before it is determined to place him in a profession, in which if he fail, it is at the expence of the health and happiness of those around him.' "—*Parkinson's Hospital Pupil*, p. 24.

---

*Oration delivered before the Fellows of the Medical Society of London, and published at their request.* By Richard Saumarez, Esq. 8vo. pp. 93. Underwood, Fleet-street.

In this Address, the ingenious author has pursued that train of inquiries for which on former occasions he has often signalized himself, and which are more amply detailed in his System of Physiology, and in his late work "On the Principles of Physiological and Physical Science." In the course of this Oration Mr. Saumarez has taken considerable pains to confute the errors of Sir Isaac Newton, whose system he contends is not founded on fact. The ignorance of Sir Isaac, and his followers, appears to Mr. Saumarez to have arisen from their erroneous mode of proceeding. Instead of explaining the phenomena of nature according to the mode and manner in which Nature herself describes them; instead of adapting the rules, or laws, to the phenomena, the phenomena have been adapted to the rules. Artificial phenomena, effected and produced by unnatural and factitious means, have been assumed for principles of physical science. A condition of things has been required, which in nature does not exist, but which must, nevertheless, be presupposed. As this subject was noticed in our analysis of the treatise on Physiological Science, we shall not detain our readers with a repetition of the arguments urged by Mr. Saumarez in support of his opinions, but rather select a new object, upon whom he has animadverted with a degree of severity that would seem to result purely from religious zeal, and not, as some would insinuate, because Mons. Richerand was a rival, and also a successful physiologist.

After explaining the nature of matter, which the orator divided into *dead*, *living*, and *common*; showing us how to distinguish between vegetables and animals, and brutes and the human species; informing us what life is, and defining man to be "*a rational soul in an animal body, which it employs as its instrument;*" Mr. Saumarez observed (and we regret our limits will not admit our detailing the facts on which his reasoning is founded), "If these truths appear, as to well-ordered minds they must appear, plain and even palpable, it will be necessary for me to show, that in attacking opposite principles I am not fighting with shadows, or exposing errors that have been long exploded: they are not only in actual existence, but are in full vigour, and at this moment are spreading their pernicious influence; in proof of which I refer to a book on this particular subject; a book, which has been translated from the French into English by Dr. De Lys and Mr. Kerrison; which has already gone through five editions, the merits of which have been proclaimed in our reviews; which, I understand, is strongly recommended by some of those who may be considered the highest authorities in their profession, and is in general circulation among students; I mean RICHERAND'S ELEMENTS OF PHYSIOLOGY."

"Instead of supposing that there exists a rational principle in man, whose tendency, when properly directed, is ever to obtain a paramount command over the organs of sense, Richerand, on the contrary, expressly declares 'that our physical (bodily) holds our moral nature under a strict and necessary dependance (subordination); that our vices and and our virtues, sometimes produced, and often modified by social education, are frequently too the result of organization;' 'that so absolutely is sensation the source of all our knowledge, that even the measure of the understanding is according to the number and perfection of the organs of sense; and that, by successively depriving them of the intelligent being, we should lower at each step his intellectual nature, whilst the addition of a new sense to those we now possess, might lead us to a multitude of unknown sensations and ideas, and would disclose to us in the beings we are concerned with, a vast variety of new relations, and would greatly enlarge the sphere of our intelligence.' From this doctrine Mr. Saumarez entirely dissents, and affirms, on the contrary, "that the organs of sense are far more perfect in those animals that have the least understanding, than they are in those which are blessed with the greatest portion."

"The organs of sense are far more perfect in brutes than they are in man; in savage than in intellectual life; in youth, than in old age. It is but justice to Richerand to say, that he is not the inventor, he is the mere propagator of this error. It emanates from that brutal system of philosophy, whose incessant object is, to elevate and to humanize the brute, whilst it degrades and brutalizes the man; or, in other words, which brutalizes man without humanizing the brute; which makes external sensation to be every thing, and internal consciousness to be nothing: whilst it encourages the improvement and the gratification of the [illegible]es, it neglects the cultivation of those more pure and noble pursuits which result from the energy of the soul. Whilst this system of false philosophy makes man a sensual, it prevents him from becoming an intellectual being. Instead of directing the mind to internal consciousness and meditation, it leads his thoughts out of himself, and directs its pursuits to the external world, of which he supposes himself the chief supreme. Intead of worshipping with humility, as a being dependent and accountable, the God of Nature, he contents himself with proudly and ignorantly exploring the external works of nature.

"It were devoutly to be wished that these important truths could be the means of inducing some of the teachers in our philosophical schools to alter their present plan. Instead of the multitude of zealous and intelligent youths being taught the principles of materialism, which unfortunately too often grow with their growth, and strengthen with their strength, we might cherish the hope of seeing them learn and understand, at a period of their lives when precepts have their greatest influence, the nature and end of their existence, the moral duties which they are to perform, the adoration which the creature owes to his Creator. If these precepts, which naturally flow from true principles, were duly impressed on the minds of pupils, every lecture of anatomy and of physiology, in the words of Galen, would prove a hymn of thanksgiving to the Almighty.

"I have searched, but alas! I have searched in vain, in this large

 and and thick-set volume, for some short passage which alluded to some of these important subjects; but I read of nothing else than of the affections of the soul, considered to be synonymous to the passions; the words are "*the Affections of the Soul, or the Passions.*" Nothing of those affections of the soul proclaimed, which flow from the love and fear of God, and from the influence of the Holy Spirit, on those who are pure of heart. None of those religious principles detailed, which excite and produce the performance of our moral duties with cheerfulness and contentment. None of those moral duties recommended, which bind by indissoluble ties, not only man to man, but the different members of which human society is composed. I, on the contrary, every where see, and deplore to see, the natural consequences which ensue from the principles assumed. Whilst I behold man made to resemble a beast, I have also to complain that the woman, instead of being the comfort and the comforter, the pattern and the partner of man, is supposed to be designed to answer the same purposes only, as those of a terrier bitch in a dog-kennel!!!"

If Mr. Saumarez has succeeded in destroying the strong hold of materialism, he has been yet more successful in establishing a claim to eloquence; and, in confuting Richerand's definition of courage, has ingeniously introduced the names of Hawke, of Nelson, and of Kutusow. The French philosopher, it seems, has stated that "*Courage arises out of a consciousness of strength;*" to which Mr. Saumarez, with a warmth and rapidity of style characteristic of an oration, replies,

"Was it, I would ask, a consciousness of physical or bodily strength, which impelled the multitude of heroes of which our proud country has to boast, to perform deeds of bravery and of glory? that impelled the veteran Hawke to be always searching for the enemy, always asking where are they? never, how many are they?

"Was it the feeble, the emaciated, the mutilated body of Nelson that led his intrepid soul to court all dangers in that glorious career of honour and of fame, in which he so often bled, and at length so nobly died?

"Shall I speak of that old octogenarian, the venerable Kutusow, who is pressed down with bodily infirmity and weakness, not more the result of original imperfection in his physical constitution, than from the various lacerations which he has sustained in fighting the enemies of his country—a hero of the first order, by whose valor and skill such deadly blows have been inflicted on the grand army of one of the greatest tyrants that ever desolated the world; the natural growth of a state of manners and of principles, such as I have been exposing?"

Having severely castigated Mons. Richerand for his morals, his religion, and his experiments upon animals, Mr. Saumarez concludes with the following opinion of his celebrated work on physiology.

"For the present, here I must stop with Mons. Richerand—with this galimatias—this hodge-podge—this noted book made up of scraps and and shreds—of odds and ends patched and stitched up together, without method or arrangement—without beginning, middle, or end; insomuch that, taken as a whole, it is as unfit to instruct the student in physiology, as the subject of it is beyond the abilities of its author."

Our readers, from the preceding samples, must now be enabled to appreciate for themselves the value of this oration; and estimate the zeal of the author, in his endeavours to promote science and establish truth.

---

*An Essay on the Philosophy, Study, and Use, of Natural History. By* CHARLES FOTHERGILL. 12mo. White and Cochrane, 1813.

Natural History is much talked of in this country, and little understood. From their broad and liberal education, medical men especially, are likely to know more of it than any other description of persons; but, as might be expected, after having acquired the general principles of natural science, they have chiefly directed their attention to chemistry and botany, thus leaving a very wide and even boundless field of inquiry for other cultivators. It has rarely happened that an excellent chemist, or profound botanist, has proved himself equally skilful in the other branches of natural history; or that an individual, who, like Buffon, grasped at the whole of nature, is adequate to convey instruction in every department of it. We may admire the grandeur of his designs, the vast scope of his endeavours, and be dazzled at the richness of his knowledge, but we do not look to him for complete information upon any one subject; we rather choose to consult the writings of an individual, who has devoted his best days to one part of science. The several branches of natural history doubtless are connected together, and so is a pin with its head; but if, in this simple instrument, a division of labor contributes to perfection in its fabrication, how much more advantageous must it be in cultivating the vast field of nature. Now it appears to us, that the learned author of this essay has not condescended to make himself an adequate chemist, botanist, or geologist, or very conversable in comparative anatomy, but he has taken a broad, hasty, and sweeping view of nature. If he excels in any one department more than another, it would seem to be in his acquaintance with the manners and habits of birds, animals, and reptiles, of which many original, amusing, and instructive, anecdotes are detailed in this little volume, which is not presented to the public as a finished essay, but rather as a pledge of future performances; and like the dove from the ark, ascertain the state of the waters, or, in other words, to make an experiment on public opinion. A portion of the preface,

 however, however, will explain the author's own views in publishing on the present occasion.

"I am not aware that any work has been written for the express purpose of awakening general attention to what may be considered the higher objects of the study of Natural History, though such a work could scarcely fail of proving highly beneficial. Smellie, the very able and judicious translator of Buffon, hath left us some valuable remarks in this branch of philosophy; and Dr. Skrimshire, lately president of the Natural History Society of Edinburgh, has written a series of Essays, which he calls 'Introductory to the Study, &c.' But nothing seems to have been attempted likely to produce a general or powerful effect on the public mind; which is the more to be regretted at a time when so much attention is paid to mere scientific arrangement. A well-regulated system, and appropriate technical terms, are very necessary towards the perfection of the science; and the architect, who would build a lofty and magnificent edifice, must not be ignorant of his tools, nor unprovided with suitable scaffolding. But the end must not be forgotten by too long a disputation on the means of attaining it: the skeleton is necessary for the support of the body; but what are the dry bones of the valley in comparison with a living, comprehensive, and immortal, soul?

"The two small volumes of Dr. Skrimshire may be very useful to the youth of both sexes, as introductory to the science; and, in this view, they appear sufficiently clear and appropriate: yet his remarks are common-place, and his genius does not seem of that bold and original cast which is likely to lift up the veil of Nature any higher than his predecessors have done. In his first essay he makes some correct observations on the utility of the study, and alludes to familiar illustrations; but he gives us nothing new, his mind is content with walking in paths that are safe, because they have already been explored; and he seems willing to leave the vast and unknown regions of unpenetrated knowledge for more enterprising and more daring spirits.

"Such productions as Ray's Wisdom of God in the Works of Creation, Derham's Physico Theology, and Paley's Natural Theology, approach nearer to that character which is likely to produce grand and comprehensive views of the natural world, the various functions of animals, and their relations one to another; together with the duties of man in the creation, as supreme over all, than any of the works written on what is called Natural History alone, and they are consequently productive of far more general good to society. Since it is the professed object of all enlightened Naturalists to seek a more intimate acquaintance with the Deity, by studying His works, and to gain more accurate and enlarged views of the nature of existence, and all visible objects, by such an employment of their time; it is surprising that mere system makers, and compilers of dictionaries, should obtain the character of Naturalists.

"Under feelings of this nature, I have felt it my duty to say something of the pursuit in general, comprehended in wider limits than it may have been hitherto surveyed, as well in respect to the variety of objects it regards, as to its real importance to the best interests of mankind, mankind, and as a source of rational and very superior amusement; since it would ill become one who has been accustomed to consider every hour that is not appropriated to profitable thinking, or useful exertion, as lost or mispent, silently to pass over an opportunity of pointing out the value and interest of a branch of knowledge, perhaps, of all others, the most fascinating. Particularly, too, at a juncture when I am about to lay the result of many years' inquiry into subjects of this nature before the tribunal of the public.

"The desire of putting forth this little Essay, imperfect as it is, has been more strongly felt from the consideration, that in this country, at least, the study of Natural History has never been either so fashionable, or so much encouraged, as it deserves to be, amongst a people so justly celebrated for the perfection to which they have carried most of the arts, and sciences, cultivated by civilized man. I know that a few individuals have risen up amongst us, whose fame in this branch of philosophy has been established on solid grounds; and I also know that the pursuit is partially cultivated in most parts of the empire; but, in a general view, it has never been admitted to hold rank with what are deemed the higher sciences in this country; nay, it has frequently happened that its most distinguished advocates have been stigmatized as persons of weak intellects, or have suffered the opprobrium that is generally cast on persons of perverted judgment; notwithstanding the truth, that of all the sciences, when considered throughout its widest limits, that of Natural History is the most comprehensive in its nature, and the most beneficial in its effects; for it embraces the knowledge and use of every object that can be rendered cognizable to the eye, or touch of man, not on the surface of this globe only, but above it, beneath it, within it, around it, and throughout the visible universe: it may, indeed, and without injustice, lay claim to the high character of being the great parent of all other sciences, which have no materials on which to work, or speculate, but such as are gained from the empire of Nature."

The work consists of twelve chapters, of which the leading heads are,—The general nature of the pursuit; its effects on individuals, and on society. A mere acquaintance with the technical terms, and a knowledge of scientific arrangement, not the true and most important object of this study. The animal kingdom the most interesting. The nature of animals. Objections to Buffon's system. Definition of the mental faculties. State of man. Sense of pain in inferior animals; their relations with each other, and the checks which restrain their numbers within due limits. Utility of different animals. Their principle of action. Change of matter continually taking place throughout the animal, vegetable, and mineral, kingdoms, &c. &c. Our limits will admit of but one extract from this interesting work.

"Nothing can afford a finer illustration of the beautiful order and simplicity of the laws which govern the creation, than the certainty, precision, and regularity, with which the natural checks on the superabundant increase of each tribe of animals are managed; and every family family is subject to the operation of checks peculiar to that species, whatever it may be, established by a wise law of the Most High, to counteract the fatal effects that might arise from an ever-active populative principle: and it is by the admirable disposition of these checks, the contemplation of which is alone sufficient to astonish the loftiest and most comprehensive soul of man, that the whole system of animal life, in all its various forms, is kept in due strength and equilibrium.

"This subject is worthy of the naturalist's most serious consideration, as, by a few hints, I will now attempt to show.

"The power or principle of increase, in respect to animal life, being kept down to the means of subsistence;—a doctrine which has so lately, and in our own country, received such ample illustration from the pen of the enlightened Malthus;—is so incontrovertibly true, and so universal in respect to all other animals, as well as to man, that it may be considered as a remarkable circumstance it should have remained so long without illustration and application, the more especially as it is no new doctrine; for this truth has been known to the philosophers of almost every age since the days of Aristotle and Plato, though it was reserved for Malthus to collect together and condense the scattered light on this subject into one splendid and irresistible focus.

"It may be granted, however, that this excellent writer is a little too frigid and mathematical when he has occasion to touch upon human passions and their gratification; and that he is a very cool lawgiver to feelings that most men find it very difficult to put under such strict moral restraint as this great philosopher sometimes deems necessary, in order to avoid greater evils. It must not, however, be forgotten that it is mathematical law which governs the universe; and that truth is not always gratifying to us!

"But it is really surprising, in this day of increased light and knowledge, that any set of men can be found, who, making pretensions to the philosophical character, are so blind, or so foolish, as to deny the truth of the main principle,—that all animals, including the lord of the creation himself, are, in respect to numbers, kept down, as they must infallibly be, to the means of subsistence: yet many such there are. '*The great law of necessity, which prevents population from increasing in any country beyond the food which it can either produce or require, is a law so open to our view, so obvious and evident to our understandings, that we cannot for a moment doubt it.*'*

"This great law pervades and affects the whole animal creation, and so active, unwearied, and rapid, is the principle of increase over the means of subsistence, amongst inferior animals, that it is evident whole genera of carnivorous beings amongst beasts, birds, fish, reptiles, and insects, have been created for the express purpose of suppressing the redundancy of others, and restraining their numbers within proper limits. But even those checks are insufficient to restrain the effects of a too rapid populative principle in some animals,

* Malthus's Essay on the Principle of Population, &c. 8vo. 3d ed. vol. ii. p. 68.

which which have, therefore, certain destructive propensities given to them by the Creator, that operate powerfully upon themselves and their offspring, as may be particularly observed in the natural history of the *rabbit*, but which is still more evidently and strikingly displayed in the life and economy of the *rat*.

"It has been calculated, and there can be no doubt of the truth of the statement, that the astonishing number of one million, two hundred and seventy-four thousand, eight hundred and forty individuals,* may be produced from a single pair of rabbits in the short space of four years, as these animals, even in their wild state, breed seven times in a year, and generally produce eight young ones each time. They are capable of procreation at the age of five or six months, and the doe carries her burthen no longer than thirty days.

"But the principle of increase is much more powerful, active, and effective, in the common grey rat, sometimes called the Norwegian rat (*mus decumanus*, Linn.), than in any other animal of equal size.

"This destructive quadruped is continually under the *furor* of animal love. The female carries her young for one month only, and she seldom or never produces a less number than twelve, but sometimes as many as eighteen, at a litter: the medium number may be taken for an average; and the period of gestation, though of such short continuance, is confined to no particular season of the year. The embraces of the male are admitted immediately after the birth of the vindictive progeny; and it is a fact which I have ascertained beyond any doubt, that the female suckles her young ones almost to the very moment when another litter is dropping into the world as their successors.

"A celebrated Yorkshire rat-catcher whom I have occasionally employed, one day detected and killed a large female rat that was in the act of suckling twelve young ones, which had attained a very considerable growth; nevertheless, upon opening her swollen body, he found thirteen quick young, that were within a few days of their birth. Supposing, therefore, that the rat produces ten litters in the course of a year, and that no check on their increase should operate destructively for the space of four years, a number not far short of three millions might be produced from a single pair in that time!

"Now the consequences of such an active and productive principle of increase, if suffered continually to operate without check, would soon be fatally obvious. We have heard of fertile plains being devastated, and large towns undermined, in Spain, by rabbits; and even that a military force from Rome was once requested of the great Augustus† to suppress the astonishing numbers of the same animals which overran the islands of Majorca and Minorca; but, if rats were suffered to multiply without the restraint of the most powerful and positive natural checks, not only would fertile plains and rich cities be undermined and destroyed, but the whole surface of the

---

* Pennant's Quadrupeds, vol. ii. p. 104, 3d ed.; also Bewick's Quadrupeds.

† Recorded by Pliny.

earth earth in a very few years would be rendered a barren and an hideous waste, covered with myriads of famished grey rats, against which man himself would contend in vain.

"But the same Almighty Being who perceived a necessity for their existence, has also restricted their numbers within proper bounds, by creating to them many very powerful enemies, and still more effectually by establishing a propensity in themselves, the gratification of which has continually the effect of lessening their numbers, even more than any of their foreign enemies. The male rat has an insatiable thirst for the blood of his own offspring: the female, being aware of this passion, hides her young in such secret places as she supposes likely to escape notice or discovery, till her progeny are old enough to venture forth and stand upon their own energies; but, notwithstanding this precaution, the male rat frequently discovers them, and destroys as many as he can, nor is the defence of the mother any very effectual protection, since she herself sometimes falls a victim to her temerity and her maternal tenderness. It is well known that rabbits have the same trait in their character, though, perhaps, not in an equal degree of force.

"Besides this propensity to the destruction of their own offspring, when other food fails them, rats hunt down and prey upon each other with the most ferocious and desperate avidity, insomuch, that it not unfrequently happens, in a colony of these destructive animals, that a single male of more than ordinary powers, after having overcome and devoured all competitors with the exception of a few females, reigns the sole, bloody, and much-dreaded tyrant over a considerable territory, dwelling by himself in some solitary hole, and never appearing abroad without spreading terror and dismay even amongst the females whose embraces he seeks.

"In this relentless and bloody character may be found one of the most powerful and positive of the checks which operate to the repression of this species within proper bounds, a character which attaches, in greater or less degree, to the whole *mus* genus, and in which we may readily perceive the cause of the extirpation of the old black rats of England; (*mus rattus*, Linn.) for, the large grey rats having superior bodily powers united to the same carnivorous propensities, would easily conquer and destroy their black opponents wherever they could be found, and whenever they met to dispute the title of possession or of sovereignty."

